# The effect of total cholesterol/high-density lipoprotein cholesterol ratio on mortality risk in the general population

**DOI:** 10.3389/fendo.2022.1012383

**Published:** 2022-12-15

**Authors:** Dan Zhou, Xiaocong Liu, Kenneth Lo, Yuqing Huang, Yingqing Feng

**Affiliations:** ^1^ Department of Cardiology, Guangdong Cardiovascular Institute, Guangdong Provincial People’s Hospital, Guangdong Academy of Medical Sciences, Guangzhou, China; ^2^ Department of Epidemiology, Centre for Global Cardio-Metabolic Health, Brown University, Providence, RI, United States; ^3^ Department of Applied Biology and Chemical Technology, The Hong Kong Polytechnic University, Hong Kong, Hong Kong SAR, China

**Keywords:** total cholesterol/high-density lipoprotein cholesterol ratio, all-cause mortality, cardiovascular mortality, nonlinear association, prognostic capacity

## Abstract

**Background:**

The relationship between the total cholesterol/high-density lipoprotein cholesterol (TC/HDL-C) ratio and all−cause and cardiovascular mortality has not been elucidated. Herein, we intend to probe the effect of the TC/HDL-C ratio on all-cause and cardiovascular mortality in the general population.

**Methods:**

From the 1999–2014 National Health and Nutrition Examination Surveys (NHANES), a total of 32,405 health participants aged ≥18 years were included. The TC/HDL-C levels were divided into five groups: Q1: <2.86, Q2: 2.86–3.46, Q3: 3.46–4.12, Q4: 4.12–5.07, Q5: >5.07. Multivariate Cox regression models were used to explore the relationship between the TC/HDL-C ratio and cardiovascular and all-cause mortality. Two−piecewise linear regression models and restricted cubic spline regression were used to explore nonlinear and irregularly shaped relationships. Kaplan–Meier survival curve and subgroup analyses were conducted.

**Results:**

The population comprised 15,675 men and 16,730 women with a mean age of 43 years. During a median follow-up of 98 months (8.1 years), 2,859 mortality cases were recorded. The TC/HDL-C ratio and all-cause mortality showed a nonlinear association after adjusting for confounding variables in the restricted cubic spline analysis. Hazard ratios (HRs) of all-cause mortality were particularly positively related to the level of TC/HDL-C ratio in the higher range >5.07 and in the lower range <2.86 (HR 1.26; 95% CI 1.10, 1.45; HR 1.18; 95% CI 1.00, 1.38, respectively), although the HRs of cardiovascular disease mortality showed no difference among the five groups. In the two-piecewise linear regression model, a TC/HDL-C ratio range of ≥4.22 was positively correlated with cardiovascular mortality (HR 1.13; 95% CI 1.02, 1.25). In the subgroup analysis, a nonlinear association between TC/HDL-C and all-cause mortality was found in those aged <65 years, men, and the no lipid drug treatment population

**Conclusion:**

A nonlinear association between the TC/HDL-C ratio and all-cause mortality was found, indicating that a too-low or too-high TC/HDL-C ratio might increase all-cause mortality. However, for cardiovascular mortality, it does not seem so. The cutoff value was 4.22. The individuals had higher cardiovascular mortality with a TC/HDL-C ratio >4.22.

## Introduction

Cardiovascular disease and cancer are the primary causes of mortality worldwide. A report from America shows the United States see 1 million deaths from cardiovascular disease per year (1). Cholesterol contributes significantly to cardiovascular disease and cancer (2, 3). Impaired intracellular cholesterol metabolism is related to the procedure of many diseases (4).

Although low-density lipoprotein cholesterol (LDL-C) level is used as the primary target of therapy, the risk of cardiovascular disease among statin-treated individuals remains high and not fully explained. Other lipid profiles may interpret some causes of the risk of cardiovascular disease and mortality. Guidelines recommend consideration of lipoprotein ratios [total cholesterol (TC)/high-density lipoprotein cholesterol (HDL-C)] in the management of cardiovascular risk (5). Previous studies have indicated a link between the TC/HDL-C ratio and cardiovascular events; however, findings from these studies have been controversial due to partially inconsistent results (6, 7). The TC/HDL-C ratio remains to be related to cardiovascular mortality among statin-treated individuals; patients in the highest range >2.83 have an increased risk of cardiovascular mortality that is 63% higher than those in the lowest range <2.23 (8, 9). The TC/HDL-C ratio is associated with cardiovascular morbidity and mortality in the general population, independently of triglycerides (TGs), albuminuria, and high-sensitivity C-reactive protein (10). It also has been proven to be an effective predictor of future cardiac events among healthy US women aged 45 years or older (11). Calculating the ratio TC/HDL-C can help us better judge cardiovascular risk when TC levels and HDL-C levels are difficult to determine. Exploring the relationship between TC/HDL-C and death can focus on the residual risk of death after LDL-C treatment.

However, few studies have examined the association of the TC/HDL-C ratio with all-cause mortality. The optimal range of the TC/HDL-C ratio for avoiding mortality is still unknown. Evidence from a large cohort among the general population is needed to address the knowledge gap.

## Materials and methods

### Population

**Figure 1:**
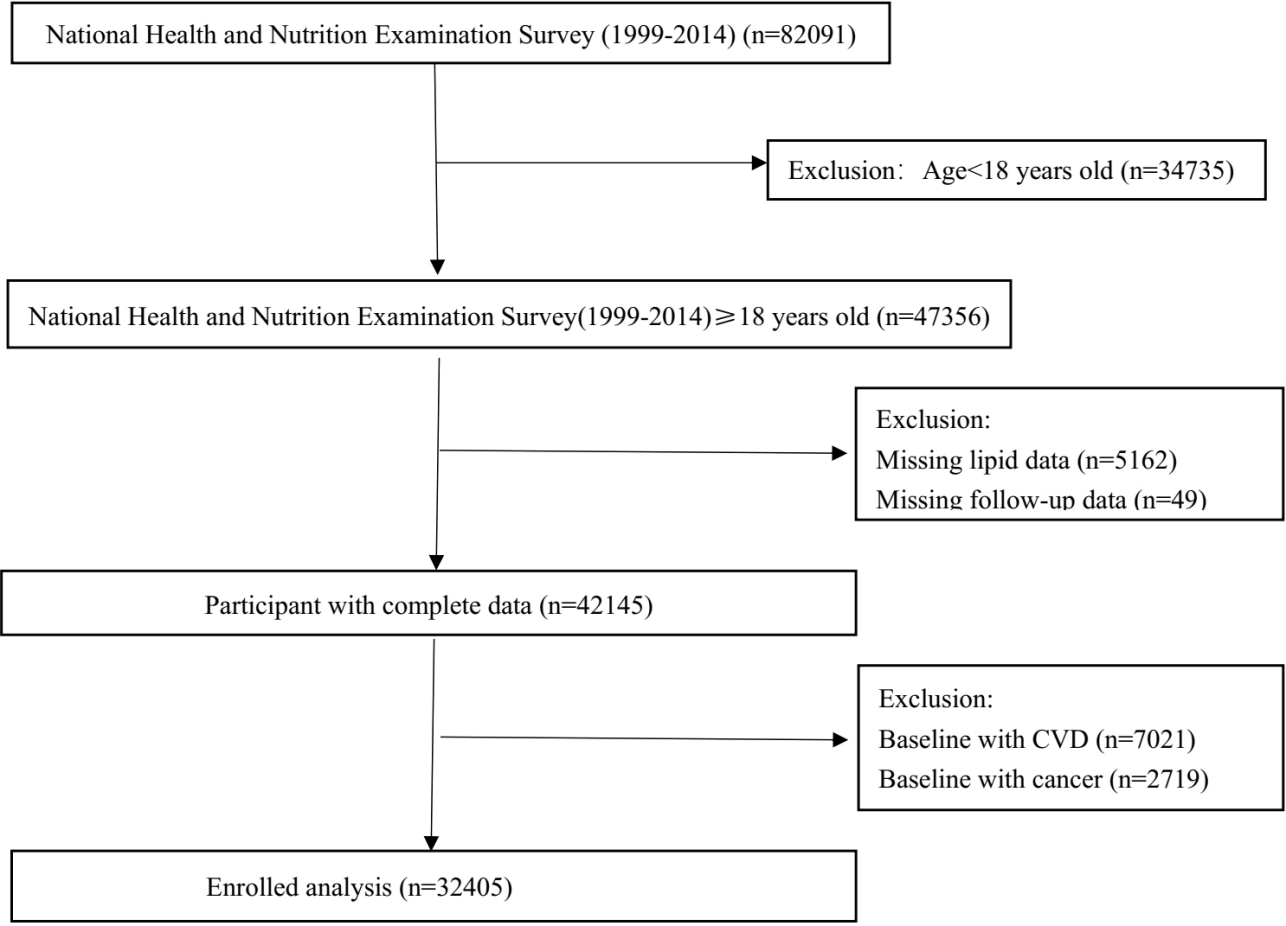
Study flowchart.

The National Health and Nutrition Examination Survey (NHANES) was a program of studies designed to assess the health and nutritional status of adults and children in the United States. Data from the NHANES for the years 1999–2014 were used for analysis, and a total of 32,405 participants aged ≥18 years with lipid data were included (**Figure 1**). From 1999 to 2014, people with cardiovascular disease (7,021 people) or cancer (2,719 people) were excluded during the baseline. The study protocol was agreed upon by the Centers for Disease Control and Prevention of the United States. All participants signed an informed consent form.

### Data collection

Demographic information was collected through questionnaires by trained personnel, including age, gender, race (white or non-white), medical history (hypertension or diabetes), and smoking status. Height, weight, and blood pressure were measured by trained personnel in a standard operating procedure. Body mass index (BMI) was calculated using weight (kg) divided by the square of height (m^2^). The estimated glomerular filtration rate (eGFR) was computed using the Modification of Diet in Renal Disease (MDRD) formula (12). Hypertension was defined as systolic blood pressure (SBP) ≥140 mmHg or diastolic blood pressure (DBP) ≥90 mmHg or self-reported history of hypertension (13). Type 2 diabetes was defined as fasting blood glucose ≥126 mg/dl (7.0 mmol/L), self-reported history of diabetes, hemoglobin A1c (HbA1C) ≥6.5%, or using hypoglycemic drugs (14).

### Lipid measurement

Lipid blood sample collection and measurement were conducted according to a standardized protocol from the Centers for Disease Control and Prevention. HDL-C was measured by direct immunoassay or by precipitation (15). Serum TC and HDL-C levels were measured enzymatically with a Hitachi 704 Analyzer (Boehringer Mannheim Diagnostics, Indianapolis, IN, USA) (16). LDL-C was measured by the Friedewald formula [LDL-C = TC – HDL-C – (TG/5)] if the TG level was ≤400 mg/dl (17).

### Clinical outcomes

Primary outcomes were all-cause mortality and cardiovascular mortality (heart disease and stroke mortality). These participants were followed up until 31 December 2015. Mortality data were extracted from the 1999–2014 NHANES public-use linked mortality files. The International Classification of Diseases, Tenth Revision, codes (I00-I09, I11, I13, I20-I51, I60-69) were used to define cardiovascular mortality.

### Statistical analysis

We applied population-weighted parametric and nonparametric tests when appropriate for exploring the associations of baseline characteristics (18). In the analysis, continuous variables were expressed as means [standard deviation (SD)] for normally distributed variables. Categorical variables were expressed as percentages (number of individuals). Baseline characteristics of participants were grouped by the TC/HDL-C ratio (Q1: <2.86, Q2: 2.86–3.46, Q3: 3.46–4.12, Q4: 4.12–5.07, Q5: >5.07). The chi-square, one-way ANOVA, Kruskal–Wallis H-test were carried out to examine the differences among these groups. Multivariate-adjusted Cox-restricted cubic spline regression was used to explore the relationship. Survival analysis was explored by using standardized Kaplan–Meier curves and log-rank tests.

Multivariate Cox regression models were conducted to examine independent factors for all-cause and cardiovascular mortality. Multivariate-adjusted Cox-restricted cubic spline regression models and a generalized additive model were used to explore the nonlinear relationship between the TC/HDL-C ratio and mortality. If nonlinear relationships were identified, a two-piecewise Cox proportional hazards model on both sides of the inflection point and log likelihood ratio test were performed. TC/HDL-C was included in the model as a continuous variable and fit a coefficient above/below cutoff value separately. We used a two-piecewise linear regression model to evaluate the nonlinear relationships between the TC/HDL-C ratio and mortality, and the optimal cutoff points were set by testing all possible values and selecting the cutoff values with the highest likelihood. Subgroup analysis was performed. All analyses were performed with R version 3.6.3 (R Foundation for Statistical Computing, Vienna, Austria), with statistical significance being identified at the level of P < 0.05.

## Results

### Baseline characteristics

The baseline characteristics according to the TC/HDL-C ratio groups were presented in **Table 1**. In total, 32,405 participants were included in this analysis with mean age of 43 years old. Among them, 51.63% were women, 67.69% were white, and 45.23% smoked. In addition, the proportion of participants with hypertension and diabetes was 33.22% and 10.04%, respectively. The proportion of antihypertensive drugs, hypoglycemic agents, and lipid-lowering drugs was 17.67%, 5.09%, and 8.81%, respectively. There were significant differences in age, gender, smoking, BMI, SBP, DBP, TC, eGFR, baseline proportion of diabetes, hypertension, and the use of lipid-lowering, antihypertensive, and hypoglycemic drugs among groups according to the TC/HDL-C concentrations (all P < 0.05), except race.

**Table 1:** Demographic and clinical characteristics according to the TC/HDL-C ratio quintiles.

	TC/HDL-C						
	Total	Q1	Q2	Q3	Q4	Q5	P for trend
Number	32,405	6,481	6,483	6,479	6,484	6,478	
Age, years	43.9 (0.18)	42.4 (0.31)	43.8 (0.30)	44.1 (0.24)	44.9 (0.26)	44.5 (0.24)	<0.001
Gender-female, %	51.6 (0.27)	70.6 (0.74)	62.1 (0.88)	53.1 (0.75)	42.2 (0.86)	30.4 (0.69)	<0.001
Race-white, %	67.6 (1.19)	66.6 (1.28)	67.5 (1.21)	68.0 (1.38)	66.5 (1.42)	69.5 (1.43)	0.086
Smoking, %	45.2 (0.57)	41.5 (0.82)	41.2 (0.85)	44.8 (0.87)	45.0(0.88)	53.2 (1.00)	<0.001
Body mass index, kg/m^2^	28.4 (0.07)	25.1 (0.10)	27.3 (0.11)	28.8 (0.10)	30.0 (0.11)	30.7 (0.12)	<0.001
Systolic blood pressure, mmHg	121.1 (0.18)	118.2 (0.30)	119.4 (0.31)	120.9(0.26)	122.3(0.30)	124.5 (0.33)	<0.001
Diastolic blood pressure, mmHg	71.1 (0.16)	68.5 (0.26)	69.2 (0.22)	71.0 (0.21)	72.4 (0.23)	74.3 (0.28)	<0.001
eGFR, mg/min/1.73 m^2^	88.7 (0.32)	89.9 (0.53)	88.7(0.52)	89.6 (0.56)	87.4 (0.46)	87.8 (0.44)	<0.001
Total cholesterol, mg/dl	198.3 (0.39)	173.8 (0.56)	185.7 (0.58)	194.6 (0.70)	206.4 (0.61)	230.5 (0.74)	<0.001
HDL cholesterol, mg/dl	52.8 (0.16)	71.4 (0.27)	58.8 (0.19)	51.5 (0.19)	45.3 (0.14)	37.4 (0.13)	<0.001
TC/HDL-C ratio	4.1 (0.01)	2.4 (0.00)	3.1 (0.00)	3.7 (0.00)	4.5 (0.00)	6.2 (0.02)	<0.001
Comorbidities, %							
Hypertension	33.2 (0.44)	26.9 (0.70)	30.2 (0.75)	34.4 (0.80)	36.1 (0.83)	38.2 (0.87)	<0.001
Diabetes	10.0 (0.23)	6.8 (0.41)	8.0 (0.43)	9.9 (0.42)	11.5 (0.50)	13.8 (0.55)	<0.001
Treatment, %							
Antihypertensive drugs	17.6 (0.35)	14.8 (0.59)	17.7 (0.65)	19.5 (0.66)	19.2 (0.71)	17.0 (0.59)	0.002
Hypoglycemic agents,	5.1 (0.16)	4.1 (0.34)	4.5 (0.31)	5.3 (0.32)	5.3 (0.34)	5.9 (0.40)	0.001
Lipid-lowering drugs	8.8 (0.25)	9.1 (0.43)	10.5 (0.56)	10.2 (0.51)	8.3 (0.42)	5.8 (0.37)	<0.001
Outcomes, %							
Cardiovascular disease mortality	1.0(0.06)	0.88(0.11)	0.8 (0.12)	1.1(0.13)	1.0 (0.12)	1.3(0.14)	0.008
All-cause mortality	6.1 (0.19)	5.6 (0.31)	5.5 (0.29)	5.7 (0.33)	6.2 (0.33)	7.3 (0.35)	<0.001

Results are mean (SD) or percentage (number of individuals).

Abbreviations: Q, quintiles; TC/HDL-C, total cholesterol/high-density lipoprotein cholesterol; eGFR, estimated glomerular filtration rate; Q1, <2.86; Q2, 2.86–3.46; Q3, 3.46–4.12; Q4, 4.12–5.07; Q5,>5.07.

### Incidence of cardiovascular and all-cause mortality

During a median follow-up of 98 months (8.1 years), 2,859 mortality cases occurred; 551 mortality cases were due to cardiovascular disease. The incidence rate of all-cause and cardiovascular mortality among the TC/HDL-C groups was shown in **Table 1**.

### TC/HDL-C ratio and all-cause or cardiovascular mortality

**Figure 2:**
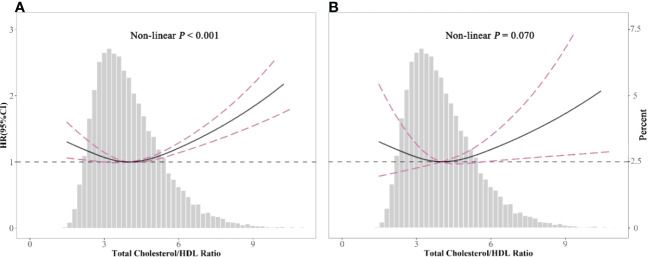
Spline analyses of all-cause **(A)** and cardiovascular **(B)** mortality by the total cholesterol/high-density lipoprotein cholesterol (TC/HDL-C) ratio in the overall cohort, and the probability distribution histogram is represented in the background. (Spline analyses were adjusted for age, gender, race, smoking, body mass index, systolic blood pressure, estimated glomerular filtration rate, diabetes and hypertension, antihypertensive drugs, hypoglycemic agents, and lipid-lowering drugs.).

**Figure 3:**
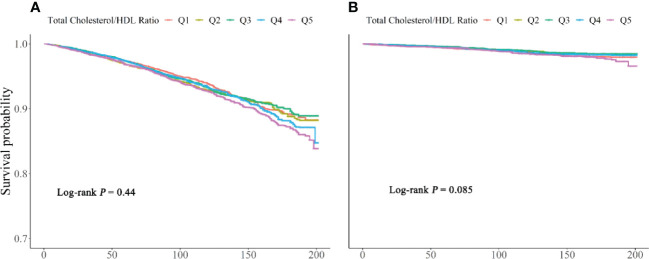
Kaplan–Meier survival curves for all-cause **(A)** and cardiovascular **(B)** mortality between Q1, <2.86, Q2, 2.86–3.46, Q3, 3.46–4.12, Q4, 4.12–5.07, and Q5, >5.07.

**Table 2** shows the estimated hazard ratio (HR) and confidence intervals (CIs) of all-cause and cardiovascular mortality according to the different TC/HDL-C ratio groups. When compared to the reference group (TC/HDL-C ratio: 3.16–3.78) in model III, the multivariable-adjusted HRs for all-cause mortality was 1.26 (1.10, 1.45) for group 5 (P < 0.05). HRs for all-cause mortality in the first group was 1.18 (1.00, 1.38). Among the large sample size of 32,405 participants, only 15,106 participants have the data for LDL-C. LDL-C has a strong relationship with cardiovascular risk. We tried to include LDL-C in the model of the 15,106 participants. The results showed severe collinearity between LDL-C and TC (**Supplementary Table S1**). LDL-C and TC showed a strong correlation (**Supplementary Table S2**). We adjusted TC in model 3, and LDL-C was excluded from the analysis. Multivariate-adjusted Cox-restricted cubic spline regression models and a generalized additive model were used to explore the nonlinear relationship between the TC/HDL-C ratio and mortality. Multivariate-adjusted Cox-restricted cubic spline regression was shown in **Figure 2**. After adjusting for some potential confounders, the relationship between TC/HDL-C and all-cause mortality was revealed to be U-shaped (P < 0.001) (**Figure 2A**), as both low and high concentrations were associated with high all-cause mortality risk. However, the nonlinear association between TC/HDL-C and cardiovascular mortality appeared to be not significant (P = 0.07) (**Figure 2B**). The results of the two-piecewise linear regression model between TC/HDL-C and mortality were demonstrated in **Table 3**. After adjusting for potential confounders, the cutoff value of all-cause and cardiovascular mortality was 3.66 and 4.22, respectively. More or less than 3.66 was related to a higher risk of all-cause mortality (all P < 0.05). When the TC/HDL-C ratio was >4.22, the association was significantly positive for cardiovascular mortality (P < 0.05). A TC/HDL-C ratio increase of 1 SD leads to a 13% risk increase for cardiovascular mortality. The cumulative survival probability of all-cause (**Figure 3A**) and cardiovascular mortality (**Figure 3B**) among the participants as stratified by TC/HDL-C levels was demonstrated in **Figure 3**. There were no significant differences among the five groups (all log-rank P > 0.05).

**Table 2:** Multivariate Cox regression analysis of the TC/HDL-C ratio with all-cause mortality and cardiovascular mortality.

	Event rate/1,000 person-years	Model I HR (95% CI), P-value	Model II HR (95% CI), P-value	Model III HR (95% CI), P-value
All-cause mortality				
TC/HDL-C ratio quintiles				
Q1	10.57	1.07 (0.93, 1.22), 0.3449	1.28 (1.12, 1.47), 0.0002	1.18 (1.00, 1.38), 0.0534
Q2	10.21	1.01 (0.88, 1.16), 0.9124	1.08 (0.95, 1.23), 0.2417	1.06 (0.92, 1.22), 0.3976
Q3	10.43	Ref	Ref	Ref
Q4	11.17	1.08 (0.93, 1.25), 0.3325	1.04 (0.89, 1.20), 0.6286	1.05 (0.90, 1.23), 0.5207
Q5	11.37	1.19 (1.04, 1.37), 0.0120	1.24 (1.09, 1.40), 0.0007	1.26 (1.10, 1.45), 0.0010
P for trend		0.0216	0.5215	0.1408
Cardiovascular mortality				
TC/HDL-C ratio quintiles				
Q1	2.07	0.85 (0.60, 1.21), 0.3676	1.04 (0.72, 1.51), 0.8255	1.06 (0.71, 1.57), 0.7853
Q2	1.60	0.78 (0.54, 1.13), 0.1959	0.84 (0.58, 1.23), 0.3744	0.94 (0.64, 1.38), 0.7507
Q3	2.09	Ref	Ref	Ref
Q4	2.20	0.92 (0.66, 1.29), 0.6386	0.89 (0.64, 1.24), 0.4807	0.86 (0.62, 1.19), 0.3603
Q5	2.38	1.15 (0.85, 1.56), 0.3645	1.23 (0.92, 1.66), 0.1673	1.09 (0.78, 1.53), 0.6111
P for trend		0.0324	0.1332	0.7329

HR, hazard ratio; CI, confidence interval; Q, quintiles; Q1, <2.86; Q2, 2.86–3.46; Q3, 3.46–4.12; Q4, 4.12–5.07; Q5, >5.07.

Model I adjusted for none.

Model II adjusted for age, gender, and race.

Model III adjusted for age, gender, race, smoking, body mass index, systolic blood pressure, estimated glomerular filtration rate, total cholesterol, comorbidities (diabetes and hypertension), and medicine use (antihypertensive drugs, hypoglycemic agents, and lipid-lowering drugs).

**Table 3:** The results of two-piecewise linear regression model between the TC/HDL-C ratio and all-cause mortality and cardiovascular mortality.

	All-cause mortalityHR (95% CI) P-value	Cardiovascular mortalityHR (95% CI) P-value
Cutoff value	3.66	4.22
<Cutoff value	0.84 (0.75, 0.93) 0.001	0.91 (0.75, 1.10) 0.331
≥Cutoff value	1.13 (1.09, 1.17) <0.001	1.13 (1.02, 1.25) 0.015
P for log likelihood ratio test	<0.001	0.076

HR, hazard ratio; CI, confidence interval.

The two-piecewise linear regression model was adjusted for age, gender, race, smoking, body mass index, systolic blood pressure, estimated glomerular filtration rate, total cholesterol, comorbidities (diabetes and hypertension), and medicine use (antihypertensive drugs, hypoglycemic agents, and lipid-lowering drugs).

We analyzed the association of the TC/HDL-C ratio and cancer mortality. **Supplementary Table S3** showed HR and 95% CI of the multivariable Cox regression model. The patients with the highest TC/HDL-C in the Q5 group had HRs ranging from 0.99 to 2.04 compared with patients in the Q3 group. It seems that a higher TC/HDL-C was related to cancer mortality, consistent with cardiovascular mortality. However, due to the very few outcomes of cancer mortality, the P-value was not significant.

### Subgroup analyses

**Figure 4:**
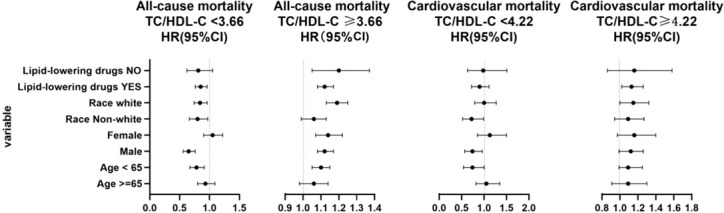
Subgroup analysis of the total cholesterol/high-density lipoprotein cholesterol (TC/HDL-C) ratio based on cutoff value. When analyzing a subgroup variable, age, gender, race, smoking, body mass index, systolic blood pressure, estimated glomerular filtration rate, total cholesterol, comorbidities (diabetes and hypertension), and medicine use (antihypertensive drugs, hypoglycemic agents, and lipid-lowering drugs) were adjusted except the variable itself.

Subgroup analysis was presented in **Figure 4**. After adjusting for some confounders, the results showed differences in the subgroups. The two-piecewise linear relationship between TC/HDL-C and all-cause mortality was significant in the populations that were aged <65 years, men, not taking lipid-lowering drugs, and no matter white or not. For those treated with lipid-lowering drugs, above the cutoff value had shown a significant association with all-cause mortality. Although a single lipid variable controlled well, the TC/HDL-C ratio contributed to additional lipid evaluation value. The two-piecewise linear relationship between TC/HDL-C and cardiovascular mortality was not significant in the subgroup.

## Discussion

The principal finding of this study was that the TC/HDL-C ratio had a nonlinear connection with all-cause mortality but not cardiovascular mortality. For cardiovascular mortality, the TC/HDL-C ratio >4.22 had higher cardiovascular mortality.

Abnormal blood lipid metabolism is a necessary condition for the occurrence of atherosclerosis. TC, LDL-C, and TG are the most often evaluated in clinical work. However, a single lipid index has been shown to be poorly predictive of cardiovascular disease. Among the patients treated with a statin, lipoprotein ratios provided additional value. In a cohort study (10), a high TC/HDL-C ratio was associated with a higher risk of cardiovascular and malignancy mortality in participants without previous cardiovascular disease and who did not use lipid-lowering drugs initially. Another analysis from the Atherosclerosis Risk in Communities (ARIC) study (19), a large cohort of participants free from atherosclerotic cardiovascular disease (ASCVD) at baseline and followed up for more than 20 years with five visits, indicated that those with a TC/HDL-C ratio ≥4.2 had a higher risk of ASCVD, independent of other clinical risk factors and the use of lipid-lowering medications. These two studies are extensive cohort studies with an extended follow-up time; our finding was consistent with these results. The ARIC study recruited participants from 1987 to 1989 in the United States from four communities, almost the same as ours; the risk of cardiovascular mortality increased for a TC/HDL-C ratio ≥4.22. However, NHANES was a program of studies conducted in the whole United States. Data from 1999 to 2014 were used. The results of our study would be more suitable nowadays. A 1 SD increase in the TC/HDL-C ratio resulted in a 13% increased risk of cardiovascular mortality. The proportion of participants with hypertension and diabetes was only 33.22% and 10.04%, respectively. The mean age was 43 years old, and the mean SBP and DBP were normal. Therefore, the population was less at risk for cardiovascular mortality. This may be the reason that those with a TC/HDL-C ratio <4.22 showed no significance. In addition, we did not distinguish coronary heart disease mortality and cerebrovascular disease mortality from cardiovascular mortality. The adjustment of different confounding factors may also have a certain effect on the results.

Our study found that the TC/HDL-C ratio had a nonlinear association with all-cause mortality. Both extremely high and low ratios indicated a high risk of all-cause mortality in populations that were aged <65 years, men, white, and not taking lipid-lowing drugs. A retrospective study (20) from China has shown that TC/HDL-C ≥3.37 had a predictive value for mortality. Moreover, a U-shaped relationship was found between TC and all-cause mortality in the general Korean population regardless of sex and age (21) and patients with type 2 diabetes mellitus (22). The association between HDL-C concentrations and all-cause mortality was U-shaped for both men and women, with both extremely high and low concentrations being associated with high all-cause mortality risk in the Copenhagen City Heart Study and the Copenhagen General Population Study (23). Our study first showed a U-shaped association between the TC/HDL-C ratio and all-cause mortality in the general population. In the follow-up, 2,859 individuals died in our research. However, 694,423 individuals died in the general Korean population study during follow-up. In addition, 5,619 men died and 5,059 women died in the Copenhagen City Heart Study and the Copenhagen General Population Study. Fewer outcomes and fewer gaps among groups meant the cumulative survival probability analysis showed no difference in all-cause mortality and cardiovascular mortality.

TC and TC/HDL-C showed a moderate correlation in our report. Although the treatment for lowering TC and LDL-C was ongoing for reducing cardiovascular events, the incidents of cardiac events are still high. A single lipid index is poorly predictive of cardiovascular disease. Among the patients treated with a statin, lipoprotein ratios provide additional value. In the report by Beale et al. (24), TC was U-shaped associated with mortality in the no lipid drug population; in the lipid drug-treated population, TC showed no difference between groups. However, our results showed after adjusting TC that the TC/HDL-C ratio had additional clinical value in the population treated with lipid-lowering drugs. A higher TC/HDL-C ratio in treated patients still had a higher risk of all-cause mortality, not cardiovascular mortality. Maybe the decreased TC reduced the artery atherosclerosis of the heart and brain; however, the TC/HDL-C ratio influenced all-cause mortality with other mechanisms. The results make us pay attention to lipoprotein ratios in all-cause mortality.

In the subgroup, a two-piecewise linear connection with all-cause mortality only had significance in the populations that were aged <65 years, men, not taking lipid-lowering drugs, and white or not white. It seems that atherosclerosis caused by cholesterol was not the leading cause of mortality in these populations, with extreme control of the TC/HDL ratio for avoiding all-cause mortality and remote control of the TC/HDL ratio for avoiding cardiovascular mortality. The population aged ≥65 years may have many comorbidities, such as hypertension, diabetes, or hyperuricemia. Recently, older women with a higher risk of heart failure with preserved ejection fraction (HFpEF) had been identified by several studies (24). Then, more risk factor management is needed to focus on these populations, not only the TC/HDL-C ratio.

There are several limitations to the study. First, the population-based sampling of NHANES permitted our analyses to represent men and women living in the United States. Second, we did not show time-fixed and time-varying follow-up lipid data. Third, despite adjusting for known or hypothesized variables to influence or confound the TC/HDL-C ratio and mortality relationship, we cannot exclude the possibility of residual confounding by unmeasured factors, such as inflammation markers, physical markers, physical activity, and uric acid.

## Conclusions

In summary, the TC/HDL-C ratio had a nonlinear connection with all-cause mortality but not with cardiovascular mortality. The cutoff value was 4.22. Individuals had higher cardiovascular mortality with a TC/HDL-C ratio >4.22. The prognostic capacity of the TC/HDL-C ratio provides complementary tools to assess the deleterious health effects of dysfunctional lipid composition. For statin-treated patients, the TC/LDL-C ratio contributes more value than TC does.

## Data availability statement

The datasets presented in this study can be found in online repositories. The names of the repository/repositories and accession number(s) can be found in the article/supplementary material.

## Ethics statement

The studies involving human participants were reviewed and approved by Protocol #2018-01. The patients/participants provided their written informed consent to participate in this study.

## Author contributions

DZ, YF contributed to the conception and design of the study. DZ drafted the manuscript. XL contributed to the acquisition of data, interpretation of data, and analysis of data. KL, YH contributed to the interpretation of data and critical revision of the article for important intellectual content. All authors contributed to the article and approved the submitted version.

## References

[1] Benjamin EJ Virani SS Callaway CW Chamberlain AM Chang AR Cheng S. Heart disease and stroke statistics-2018 update: A report from the American heart association. Circulation (2018) 137(12):e67–e492. doi: 10.1161/CIR.0000000000000558 29386200

[2] Lin X Liu L Fu Y Gao J He Y Wu Y. Dietary cholesterol intake and risk of lung. Cancer: A Meta-Analysis[J]. Nutrients (2018) 10(2):185. doi: 10.3390/nu10020185 PMC5852761 29419756

[3] Berger S Raman G. Dietary cholesterol and cardiovascular disease: a systematic review and meta-analysis. Am J Clin Nutr (2015) 102(2):276–94. doi: 10.3945/ajcn.114.100305 26109578

[4] Radhakrishnan A Rohatgi R. Cholesterol access in cellular membranes controls hedgehog signaling. Nat Chem Biol (2020) 16(12):1303–13. doi: 10.1038/s41589-020-00678-2 PMC7872078 33199907

[5] Genest J Frohlich J Fodor G McPherson R. Recommendations for the management of dyslipidemia and the prevention of cardiovascular disease: summary of the 2003 update. Cmaj (2003) 169(9):921–4. doi: 10.1016/j.chiabu.2006.02.015 PMC219626 14581310

[6] Sniderman AD Jungner I Holme I Aastveit A Walldius G. Errors that result from using the TC/HDL c ratio rather than the apoB/apoA-I ratio to identify the lipoprotein-related risk of vascular disease. J Intern Med (2006) 259(5):455–61. doi: 10.1111/j.1365-2796.2006.01649.x 16629851

[7] Kastelein JJ van der Steeg WA Holme I Gaffney M Cater NB Barter P. Lipids, apolipoproteins, and their ratios in relation to cardiovascular events with statin treatment. Circulation (2008) 117(23):3002–9. doi: 10.1161/CIRCULATIONAHA.107.713438 18519851

[8] Mora S Glynn RJ Boekholdt SM Nordestgaard BG Kastelein JJ Ridker PM. On-treatment non-high-density lipoprotein cholesterol, apolipoprotein b, triglycerides, and lipid ratios in relation to residual vascular risk after treatment with potent statin therapy: JUPITER (justification for the use of statins in prevention: an intervention trial evaluating rosuvastatin). J Am Coll Cardiol (2012) 59(17):1521–8. doi: 10.1016/j.jacc.2011.12.035 PMC3338194 22516441

[9] Arsenault BJ Rana JS Stroes ES Després JP Shah PK Kastelein JJ. Beyond low-density lipoprotein cholesterol: respective contributions of non-high-density lipoprotein cholesterol levels, triglycerides, and the total cholesterol/high-density lipoprotein cholesterol ratio to coronary heart disease risk in apparently healthy men and women. J Am Coll Cardiol (2009) 55(1):35–41. doi: 10.1016/j.jacc.2009.07.057 20117361

[10] Kappelle PJ Gansevoort RT Hillege JL Wolffenbuttel BH Dullaart RP. Apolipoprotein B/A-I and total Holesterol/high-density lipoprotein cholesterol ratios both predict cardiovascular events in the general population independently of nonlipid risk factors, albuminuria and c-reactive protein. J Intern Med (2011) 269(2):232–42. doi: 10.1111/j.1365-2796.2010.02323.x 21129046

[11] Ridker PM Rifai N Cook NR Bradwin G Buring JE. Non-HDL cholesterol, apolipoproteins a-I and B100, standard lipid measures, lipid ratios, and CRP as risk factors for cardiovascular disease in women. Jama (2005) 294(3):326–33. doi: 10.1001/jama.294.3.326 16030277

[12] National Kidney Foundation. K/DOQI clinical practice guidelines for chronic kidney disease: evaluation, classification, and stratification. Am J Kidney Dis (2002) 39(2 Suppl 1):S1–266. doi: 10.1111/j.1745-7599.2002.tb00119.x 11904577

[13] Chobanian AV Bakris GL Black HR Cushman WC Green LA Izzo JL Jr. The seventh report of the joint national committee on prevention, detection, evaluation, and treatment of high blood pressure: the JNC 7 report. Jama (2003) 289(19):2560–72. doi: 10.1001/jama.289.19.2560 12748199

[14] American Diabetes Association. Standards of medical care for patients with diabetes mellitus. Diabetes Care (2003) 26 Suppl 1:S33–50. doi: 10.2337/diacare.26.2007.s33 12502618

[15] Bucholz EM Rodday AM Kolor K Khoury MJ de Ferranti SD. Prevalence and predictors of cholesterol screening, awareness, and statin treatment among US adults with familial hypercholesterolemia or other forms of severe dyslipidemia (1999-2014). Circulation (2018) 137(21):2218–30. doi: 10.1161/CIRCULATIONAHA.117.032321 PMC6381601 29581125

[16] Doran B Guo Y Xu J Weintraub H Mora S Maron DJ. Prognostic value of fasting versus nonfasting low-density lipoprotein cholesterol levels on long-term mortality: insight from the national health and nutrition examination survey III (NHANES-III). Circulation (2014) 130(7):546–53. doi: 10.1161/CIRCULATIONAHA.114.010001 25015340

[17] Friedewald WT Levy RI Fredrickson DS. Estimation of the concentration of low-density lipoprotein cholesterol in plasma, without use of the preparative ultracentrifuge. Clin Chem (1972) 18(6):499–502. doi: 10.1093/clinchem/18.6.499 4337382

[18] Hernaez R McLean J Lazo M Brancati FL Hirschhorn JN Borecki IB. Association between variants in or near PNPLA3, GCKR, and PPP1R3B with ultrasound-defined steatosis based on data from the third national health and nutrition examination survey. Clin Gastroenterol Hepatol (2013) 11(9):1183–1190.e1182. doi: 10.1016/j.cgh.2013.02.011 23416328 PMC4197011

[19] Quispe R Elshazly MB Zhao D Toth PP Puri R Virani SS. Total cholesterol/HDL-cholesterol ratio discordance with LDL-cholesterol and non-HDL-cholesterol and incidence of atherosclerotic cardiovascular disease in primary prevention: The ARIC study. Eur J Prev Cardiol (2020) 27(15):1597–605. doi: 10.1177/2047487319862401 PMC6952589 31291776

[20] Chen L Xu J Sun H Wu H Zhang J. The total cholesterol to high-density lipoprotein cholesterol as a predictor of poor outcomes in a Chinese population with acute ischaemic stroke. Lipids Health Dis (2017) 31(6):e22139. doi: 10.1002/jcla.22139 PMC6816911 28124804

[21] Yi SW Yi JJ Ohrr H. Total cholesterol and all-cause mortality by sex and age: a prospective cohort study among 12.8 million adults. Sci Rep (2019) 9(1):1596. doi: 10.1038/s41598-018-38461-y 30733566 PMC6367420

[22] Lee S Zhou J Leung KSK. Development of a predictive risk model for all-cause mortality in patients with diabetes in Hong Kong. BMJ Open Diabetes Res Care (2021) 9(1):e001950. doi: 10.1136/bmjdrc-2020-001950 PMC8201981 34117050

[23] Madsen CM Varbo A Nordestgaard BG Eur Heart J. Extreme high high-density lipoprotein cholesterol is paradoxically associated with high mortality in men and women: two prospective cohort studies. Eur Heart J (2017) 38(32):2478–86. doi: 10.1093/eurheartj/ehx163 28419274

[24] Beale AL Nanayakkara S Segan L Mariani JA Maeder MT van Empel V. Sex differences in heart failure with preserved ejection fraction pathophysiology: A detailed invasive hemodynamic and echocardiographic analysis. JACC Heart Fail (2019) 7(3):239–49. doi: 10.1016/j.jchf.2019.01.004 30819380

