# Supplementary material for: The effect of total cholesterol/high-density lipoprotein cholesterol ratio on mortality risk in the general population
Source: Front Endocrinol (Lausanne). 2022 Dec 15;13:1012383. doi: 10.3389/fendo.2022.1012383 (PMC9797665; doi:10.3389/fendo.2022.1012383)
Supplement: Supplementary file 1 [file Table_1.docx]

Supplement Table 1 showed the VIF in model with LDL-C or not

|  | With LDL-C | Not with LDL-C |
| --- | --- | --- |
| TC/HDL-C | 2.013624 | 1.690564 |
| age | 2.462479 | 2.48269 |
| gender | 1.883935 | 1.817541 |
| Race-white | 1.360723 | 1.346206 |
| Smoking | 1.611727 | 1.561674 |
| SBP | 1.969228 | 1.933696 |
| BMI | 2.276792 | 2.221281 |
| eGFR | 2.03264 | 1.995956 |
| TC | 7.633459 | 1.967357 |
| LDL-C | 7.965528 | - |
| T2DM | 2.106639 | 2.087517 |
| Hypertension | 3.039052 | 2.908166 |
| Lipid-lowering drugs | 1.520154 | 1.513177 |
| Antihypertensive drugs | 2.402973 | 2.273969 |
| Hypoglycemic agents | 1.819303 | 1.818728 |

Model refers the association of TC/HDL-C ratio with all-cause mortality.

Supplement Table 2 Pearson correlation analysis between TC, LDL-C and TC/HDL-C

|  | r |
| --- | --- |
| LDL-C & TC | 0.91 |
| LDL-C & TC/HDL-C | 0.62 |
| TC & TC/HDL-C | 0.47 |

Supplement Table 3 showed HR and 95%CI of multivariable cox regression model.

|  | Adjusted Model  HR (95%CI) | p-value |
| --- | --- | --- |
| TC/HDL-C ratio quintiles |  |  |
| Q1 | 0.90(0.58-1.41) | 0.644 |
| Q2 | 0.96(0.67-1.39) | 0.846 |
| Q3 | Ref |  |
| Q4 | 1.0(0.70-1.41) | 0.978 |
| Q5 | 1.42(0.99-2.04) | 0.053 |

Adjusted Model adjust for age, gender, race, smoking, body mass index, systolic blood pressure, estimated glomerular filtration rate, total cholesterol, comorbidities (diabetes and hypertension), and medicine use (antihypertensive drugs, hypoglycemic agents, and lipid-lowering drugs).
